# Phospho-specific flow cytometry for pharmacodynamic monitoring of immunosuppressive therapy in transplantation

**DOI:** 10.1186/2047-1440-1-20

**Published:** 2012-11-16

**Authors:** Carla Baan, Anne Bouvy, Ramin Vafadari, Willem Weimar

**Affiliations:** 1 Department of Internal Medicine, Erasmus MC, University Medical Center, Rotterdam, The Netherlands; 2 Department of Internal Medicine, Erasmus MC, University Medical Center Rotterdam, P.O. Box 1738, Room Ee559, Rotterdam, DR 3000, The Netherlands

**Keywords:** Immunosuppressive medication, Kidney transplantation, Regulatory T cells, Phospho-specific flow cytometry

## Abstract

Organ transplant recipients frequently suffer from toxicity or from lack of efficacy of immunosuppressive drugs, which can be attributed to individual variations in drug sensitivity. This problem can be resolved by applying pharmacodynamic monitoring that focuses on measuring the biological effects of drugs. Here we discuss the new technique called phospho-specific flow cytometry to monitor the activity of intracellular immune signaling pathways at the single-cell level in whole blood samples. Through this tool the efficacy of immunosuppressive medication can be assessed, novel targets can be identified, and differences in drug sensitivity between cells and patients can be clarified.

## Review

### Background

To prevent and to treat alloreactivity, transplant recipients are on immunosuppressive medication for life. These drugs target the immune system in a non-specific manner by affecting immune cell activation, clonal expansion and differentiation. Standard immune suppressing regimens consists of a calcineurin inhibitor (CNI, e.g., tacrolimus/cyclosporine), an inosine monophosphate dehydrogenase inhibitor (mycophenolate mofetil, MMF) and corticosteroids. The therapeutic window of CNIs is small, which places patients at risk of toxicity in case of over-dosing and rejection when under-dosed. Examples of debilitating side effects of immunosuppressive medication are infections, diabetes, nephrotoxicity and malignancies, all influencing patient and graft survival and quality of life.

Because of these debilitating side effects, there is a great need for (1) safer and more selective immunosuppressive agents and (2) better monitoring tools and parameters.

In the recent years, new pharmacologic agents have been introduced in the transplantation clinic to more specifically target the molecules of the T-cell activation cascade. Novel agents are drugs that target T-cell receptor (TCR) signaling (e.g., sotrastaurin), co-stimulation pathways (belatacept) and cytokine signaling pathways (tofacitinib). Based on the results found in experimental models, it is expected that these novel immunosuppressants are more specific than CNI.

Here we will touch briefly on the mechanisms of action of these immunosuppressants and discuss the developments in pharmacodynamic monitoring in transplantation patients.

#### T-cell activation and intracellular signaling pathways: targeting signal 1

After T-cell activation, several intracellular signaling pathways are used for proliferation, differentiation and death. This involves a cascade of phosphorylation and dephosphorylation of intracellular molecules by phosphatases (enzymes that remove phosphate groups from other proteins) and kinases (enzymes that modify proteins by adding phosphate groups).

**Figure 1:**
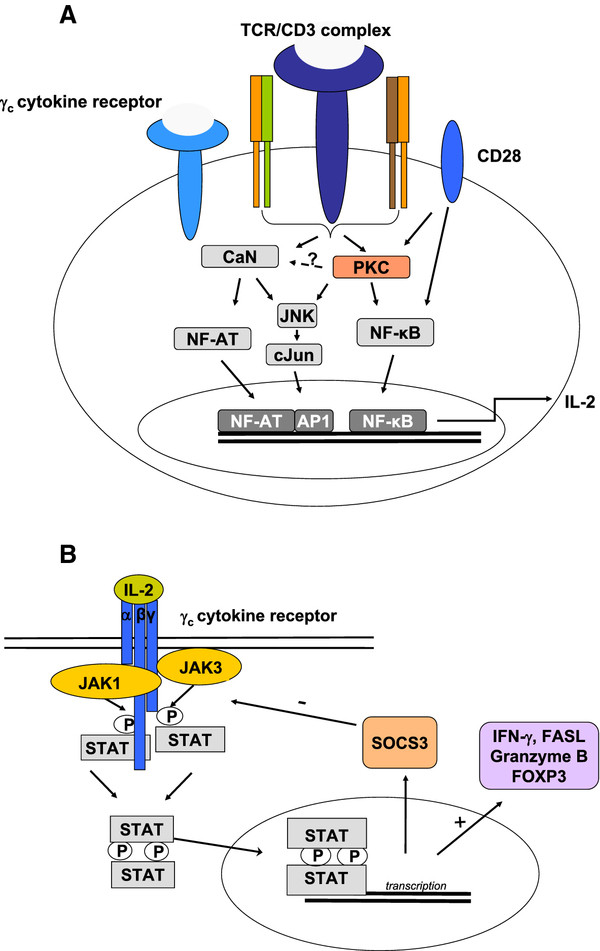
**(A) The role of PKC in TCR-CD28-induced signaling in T cells.** In response to TCR-CD28 stimulation, PKC is recruited and activated, which enables activation of JNK and NF-κB. The PKC-mediated activation of NFAT is controversial. Taken together, PKC plays the central role in the TCR-CD28-mediated induction of gene transcription, leading to proliferation and cytokine secretion in T cells. (**B**) The IL-2R consists of three subunits: α-chain (IL-2R; i.e., CD25), β-chain (IL-2Rß; CD122) and the common cytokine-receptor γ-chain (γc; CD132). Binding of IL-2 to the heterotrimer IL-2R initiates the activation of Janus-activated kinase 3 (JAK3), which associates with the γc, while JAK1 associates with IL-2Rβ. Both phosphorylate tyrosine residues in the cytoplasmic part of IL-2Rβ and the γc. Subsequently, the JAK molecules are activated, which amplifies the association of these tyrosine kinases and the signal transducer and activator of transcription 5 (STAT5) or STAT3 with the cytoplasmic tail of IL-2Rβ. Recruited STATs are phosphorylated by activated JAKs. Activated STATs translocate to the nucleus and activate gene transcription of interferons, e.g., (IFN)-γ, granzyme B, Fasligand (FasL), suppressors of cytokine signaling (SOCS) and the transcription factor for regulatory T cells: FOXP3 binding to DNA promoter sequences.

In brief, TCR activation results in the activation of tyrosine kinases of the Syk/Zap-70 family, Tec and Scr families. Upon ligand binding, Src-family kinases phosphorylate tyrosine residues located within immunoreceptor tyrosine-based activation motifs on the cytosolic side of the TCR/CD3 complex by lymphocyte protein-tyrosine kinase (Lck) followed by its phosphorylation and activation by CD45 receptor tyrosine phosphatase. Zap-70 is recruited to the TCR/CD3 complex where it becomes activated, promoting recruitment and phosphorylation of downstream adaptor or scaffold proteins. Zap-70 promotes recruitment of the molecule named Vav and phosphorylates the linker for activated T cells (LAT), a transmembrane and adaptor that links the TCR signal to many downstream events, resulting in the activation of the transcription factors, e.g., JNK, MAPK, NF-κB, AP-1 and NF-AT [1-3]. For example, NF-κB activation is promoted by protein kinase C (PKC)θ, which acts as an intermediate in the transduction of activation by TCR receptor signaling and CD28 co-stimulation [4]. NF-κB in concert with the transcription factors NF-AT and AP-1 contributes to interleukin (IL)-2 messenger RNA transcription, a key molecule in the response towards the allograft (Figure 1A).

Searching for selective immunosuppressive agents to prevent and to treat allograft rejection, phase I and II trials are running with immunosuppressive drugs that target the NF-κB pathway. Particularly, the efficacy and specificity of sotrastaurin (formerly named AEB071), a low molecular mass synthetic compound that potently inhibits all PKC isoforms is currently being studied in kidney transplant patients (Table 1). The phase II trial of sotrastaurin showed little benefit to renal graft function with an excess of acute rejection episodes, gastrointestinal disorders and serious infections when used as maintenance therapy in combination with mycophenolate mofetil and steroids [5,6].

**Table 1:** Complications after organ transplantation and therapeutic options

**Complications and immune responses**	**Identified cell type**	**Key signaling molecule(s) of**	**Therapeutic options**
Ischemia reperfusion injury (IRI)	NK and NKT cells, neutrophils, macrophages	NF-AT, NF-κB, STATs,	T-cell depletion (rATG/alemtuzumab)
		MAPK-p38, ERK, JNK	
		NF-κB, AP-1	
Hyper-acute rejection	B- and plasma cells	NF-AT, NF-κB, AKT	IvIG, T- and B-cell depletion (rATG, alemtuzumab, rituximab), bortezomib
		MAPK-p38, ERK, JNK, AP-1	
Acute rejection	Cells from the innate and adaptive immune system	NF-AT	CNI
		PKC, NF-κB	Sotrastaurin/AEB071,
		JAK1/3	tofacitinib/CP-690,550
		NF-κB	Belatacept
		mTOR	Everolimus/sirolimus
			T-cell depletion (rATG/alemtuzumab)
Chronic rejection	Memory T cells,	NF-AT, NF-κB, AKT, PI3K, MAPK-p38, ERK, JNK, AP-1	Anti-CD2 fusion protein (alefacept)
	B cells		Depletion of memory T cells (efalizumab)
Tolerance	FoxP3+ regulatory T cells	mTOR	Everolimus/sirolimus

*CNI* Calcineurin inhibitor.

*IvIG* Intravenous immune globulin.

*rATG* rabbit antithymocyte globulin.

#### Co-stimulation: targeting signal 2

The co-stimulatory signal augments T-cell activation by interactions with cell surface molecules expressed by antigen-presenting cells (APC). In this respect, augmenting interactions are: CD40/CD40ligand, CD28/CD80 and/or CD28/CD86. Inversely, T-cell activation is reduced by the co-stimulation molecule cytotoxic T-lymphocyte antigen (CTLA)-4, which transmits a T-cell inhibitory signal after binding to CD80 and/or CD86. The humanized receptor conjugate of CTLA-4 and IgG1, CTLA-4 Ig (belatacept), which indirectly blocks CD28 signaling, is approved for use in organ transplantation. Blockade of CD28 activation prevents the activation of NF-κB activation, the transcriptional target for co-stimulatory activity [7].

Two recently completed phase 3 trials of belatacept (nulojix®) in kidney transplantation, the so-called BENEFIT and BENEFIT-EXT studies, indicate that this agent is a safe and effective immunosuppressant leading to significantly better renal function (GFR, glomerular filtration rate) as compared to a cyclosporine-based regimen in kidney transplant recipients [8-10]. Acute rejection rates were higher but clinically acceptable among belatacept-treated patients (17-22% at 12 months in the belatacept groups versus 7-14% in the cyclosporine groups). While posttransplant lymphoproliferative disease was also more common in those on belatacept, this was largely limited to previously EBV seronegative recipients.

#### Cytokine signaling: targeting signal 3

The third option studied to treat organ transplant patients with more selective immunosuppressive medication is targeting signal 3: the IL-2 signaling pathway. Blockade of the IL-2 pathway prevents T-cell differentiation and production of effector molecules. This can now be achieved with the immunosuppressant named tofacitinib, formerly known as CP-690,550 and tasocitinib (Table 1). The Janus kinase (JAK)/signal transducer and activator of the transcription (STAT) signal transduction pathway is essential in transmitting cytokine-mediated signals of the IL-2 family members (IL-2, IL-4, IL-7, IL-15, IL-21) to the nucleus in order to alter gene expression programs [11]. JAKs possess two near-identical phosphate-transferring domains ( [12], Figure 1B). One domain exhibits the kinase activity, while the other negatively regulates the kinase activity of the first. The four enzymes JAK1, JAK2, JAK3 and tyrosine kinase (TYK) 2 transduce cytokine-mediated signals. After recruitment, STATs are phosphorylated by the JAKs at a specific activating tyrosine residue. This results in dissociation from the receptor, formation of STAT homo- or heterodimers, and translocation to the nucleus. Seven STATs have been identified that after activation interact with specific DNA sequences in target promoters to modify gene expression [12]. For instance IL-2 receptor signaling is mediated through activation of JAK1 and JAK3 with subsequent phosphorylation and activation of STAT3 and STAT5, which are key molecules for T-cell development and activation.

Treatment with the JAK1/3 antagonist tofacitinib in kidney transplant recipients demonstrated acceptable safety and tolerability in combination with mycophenolate mofetil [13]. Tofacitinib-based treatment resulted in relatively low acute rejection rates but was unfortunately accompanied by high infection rates suggestive of over-immunosuppression [14].

#### Rational for pharmacodynamic immunomonitoring

Overall the novel immunosuppressants currently being tested in phase II and III trials (sotrastaurin, tofacitinib) and the FDA-approved belatacept failed to show potent, selective immunosuppression. To gain insight into the mechanisms behind these unexpected side effects and the sometimes observed lack of efficacy, it is useful to perform immune monitoring. This will give answers to the questions about the selectivity and efficacy of these novel immunosuppressive compounds. By immune monitoring, questions like the following will be unraveled: “Do the agents indeed block their target(s) in vivo?” “How do naïve and antigen-experienced memory T cells behave?” “Do immunosuppressive agents influence the function of the suppressor regulatory T cells (Tregs) that control immune reactivity?” “Does redundancy in the cytokine network affect the efficacy of the studied immunosuppressant?” Recent developments in phospho-specific flow cytometry now provide the opportunity for routine measurements of intracellular signaling molecules in different T-cell subsets. In the next part of the article, we will discuss this tool with its recent developments and applications to better determine the balance between drug efficacy and side effects for the individual patient.

### Immune monitoring by classical flow cytometry

Flow cytometry is the standard immune monitoring method to measure T-, B- and NK-cell numbers in the circulation of rATG/alemtuzumab-treated patients and to verify CD25 blockade on T cells during basiliximab therapy. Reports on these studies showed correlations between cell numbers and the occurrence of opportunistic infections and the malignancies without providing insight on the function of the repopulated T cells and of T cells covered with anti-CD25 mAb [15-18]. For instance the infections in rATG-treated patients are explained by the low T-cell and NK-cell numbers. However, there is now evidence that the function of the repopulated T cells is impaired, which may affect the immunity of rATG-treated patients [17-19]. Also the role of regulatory T cells (Tregs) in clinical organ transplantation is often studied by their phenotype. One of the first papers describing a role for these cells in drug-free tolerant kidney transplant patients was by Louis et al., who reported high numbers of peripheral Tregs compared to patients with chronic rejection [20]. In line with effector T cells, also the numbers and function of Tregs are influenced by immunosuppressive drugs whereby both Treg-favoring and -hampering effects were reported [21-25]. Also, the first studies analyzing the mechanisms of action of tofacitinib and belatacept report an effect on peripheral Treg numbers. A significant decrease in Treg numbers with unexpected potent regulatory capacities of tofacitinib-treated patients was found [26]. Additional information on why the suppressive function was not influenced by tofacitinib or how rATG triggers the induction of Tregs in vivo would have been helpful to understand these findings.

Accordingly, there is a clear-cut need for methods that provide information at the molecular level of immune competent cells. A method that recently became available for clinical research and diagnostics and offers these applications is phospho-specific flow cytometry. By this method intracellular signaling pathways at the single-cell level can be measured. It is a quick and reliable method that combines the extracellular cell surface characteristics with intracellular molecular activities and thus will provide the additional information required for to better understand T-cell functions during immune responses in immunosuppressed transplantation patients.

### Phospho-specific flow cytometry: a novel tool to measure intracellular signaling pathways

With the recent advances in flow cytometry, the number of parameters that can be measured has been largely expanded. These new parameters now allow us to monitor immune responses functionally at rest and following activation at the single-cell level. This includes parameters such as ligand-induced activation, intracellular cytokine production, cytotoxic and proliferative activities, and most recently intracellular signaling pathways. The availability of phospho-specific antibodies makes it possible to study protein phosphorylation at the posttranslation level, thereby influencing the activity of intracellular proteins. Protocols have been developed that can be used to study, in whole blood, cellular activation cascades both in the presence and absence of agents that stimulate or inhibit cellular functions. This approach will provide information on the dynamics of the immune responses seen in organ transplant patients. But one should be aware that this is only the beginning of this new research area as more than 80,000 unique phosphorylations have been described in mammalian cells ( http://www.phosphosite.org).

**Figure 2:**
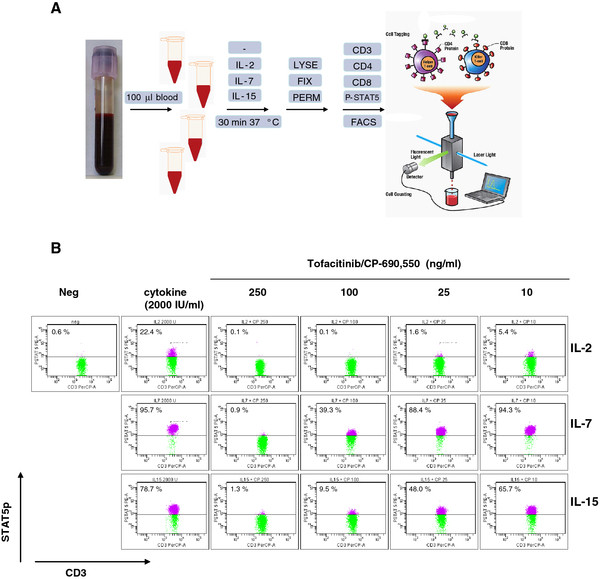
**(A) Staining procedure of STAT5. Whole blood (100 μl) is stimulated by IL-2, IL-7 or IL-15 for 30 min at 37°C.** Red blood cells are lysed and white blood cells are fixed for 10 min at 37°C with Lyse/Fix Buffer (BD Biosciences, San Jose, CA). Next, the cells are washed in FacsFlow buffer (BD Biosciences) and permeabilized with cold 70% methanol for 30 min at −20°C, washed twice in FacsFlow buffer supplemented with 0.5% bovine serum albumin followed by standard staining procedures for P-STAT5 (clone Y694), CD3, CD4 and CD8. (**B**) Whole blood stimulated for 30 min with IL-2, IL-7 and IL-15 (2,000 IU/ml) in the absence and presence of different concentrations of tofacitinib/CP-690,550. P-STAT5 expression by CD3 T cells.

**Figure 3:**
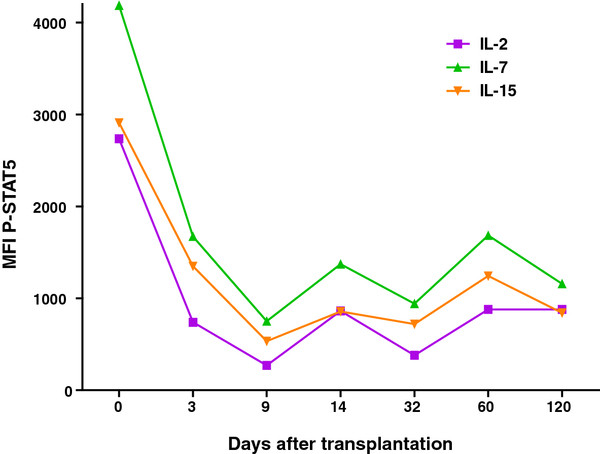
**STAT5 phosphorylation by CD4+ T cells after whole blood activation with IL-2, IL-7 and IL-15 was inhibited in this patient on tofacitinib/CP-690,550 therapy.** Cytokine-activated P-STAT5 is plotted at the Y-axis as the median fluorescence intensity (MFI) of cytokine-stimulated P-STAT5 minus the MFI of the unstimulated sample.

**Figure 4:**
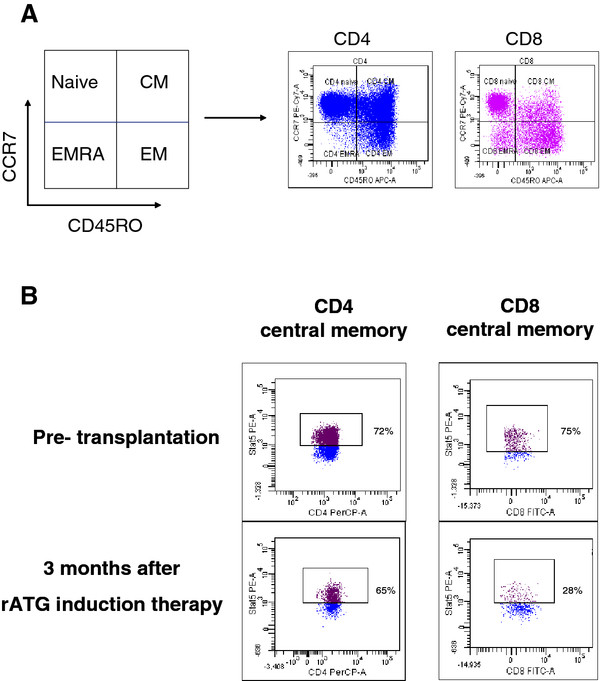
**(A) Example of the gating strategy used.** CD4+ and CD8+ T cells were analyzed for their cell surface expression of CCR7 and CD45RO to define the naïve, effector memory (EM), central memory (CM) and effector memory RA + (EMRA) T-cell subsets. (**B**) Example of decreased IL-2-induced STAT5 phosphorylation after rATG therapy in CD8 central memory T cells (CD45RO + CCR7+) and not in CD4 central memory T cells.

Key complications that occur after transplantation are ischemia reperfusion injury and acute- and chronic rejection (Table 1). The underlying mechanisms are complex, and different immune competent cells are involved that interact with each other and with the grafted tissue. Consequently, the therapeutic approach differs between these clinically relevant complications. As summarized in the table, many molecules participate in transmitting signals. The immunosuppressive agents tofacitinib and sotrastaurin were designed to specifically target these molecules, JAK1/3 and PKCθ, respectively. Others like the calcineurin inhibitors and mTOR inhibitors, both isolated from fungi, also target intracellular signaling molecules. One of the first papers showing the potential of pharmacodynamic monitoring of mTOR inhibitor molecules was recently reported [27]. By whole blood phospho-specific flow cytometry, rapamycin was found to inhibit a downstream target of mTOR: the phosphorylated S6 ribosomal protein. This assay may have advantages over the existing mTOR activity tests, which are far from reliable in predicting the efficacy of sirolimus and everolimus. Proof that indeed the analysis of the phosphorylated S6 ribosomal protein is useful for clinical purposes has not been provided yet, but the paper by Barten’s group using clinically relevant concentrations of sirolimus shows its potential for therapeutic drug monitoring. A nice example demonstrating the power of phospho-specific flow cytometry was given by Wu et al., who reported STAT5 activation in NK cells and T cells of melanoma patients who received a bolus infusion of IL-2 [28]. It was speculated that the responders and non-responders to IL-2 treatment can be identified by STAT5 measurements of peripheral blood cells. Further defective STAT1 phosphorylation was reported in lymphocytes from the majority of melanoma patients *vs.* healthy individuals after treatment with type I interferon [29]. And basal activation of STAT signaling and reduced response to type I and II interferons, IL-2, IL-6 and IL-10, may be helpful to identify the activity and severity of systemic lupus erythematosus [30]. In kidney transplant patients, we analyzed the effects of the JAK1/3 antagonist tofacitinib on IL-2-, IL-7- and IL-15-triggered activation of STAT5 and found a dose-dependent inhibition (Figure 2A and B). The whole blood analysis of tofacitinib-treated patients nicely showed inhibition of the IL-2-, IL-7- and IL-15-activated P-STAT5. An example of P-STAT5 measured in CD4+ T cells from a kidney transplant patient during tofacitinib treatment is depicted in Figure 3. Phospho-specific flow cytometry can also be used to study cross reactivity of an immunosuppressive compound. For instance, tofacitinib inhibits JAK2 activation, a pathway that plays a role in hematopoiesis and may explain the mild anemia observed in tofacitinib-treated patients [25,31]. Apart from the analysis of typical target signaling molecules, e.g., NF-κB in sotrastaurin-treated patients, mTOR in sirolimus and p38 MAPK signaling in CNI-treated kidney transplant patients, this phospho-specific flow cytometry technique can also be used to study T-cell function before and after rATG induction treatment and to further unravel the mechanism of action of immunosuppressive agents like CNI [26,32]. For instance, we used this technique to study the function of T cells after rATG induction therapy and found impaired signal 3 responses by the memory T-cell population. Particularly the CD8+ memory T cells responded poorly upon IL-2 activation by P-STAT5 (Figure 4). This finding may help to explain why rATG-treated patients often suffer from severe viral infections. It is a combination of different immune phenomena: (1). low T-cell numbers and (2) impaired T-cell function. The latter finding shows the strength of the phospho-flow technology: detection of abnormal signaling signatures as an indicator for impaired cellular function.

### Challenges to clinical practice and limitations of flow cytometry

The clinical applications for phospho-protein analysis by flow cytometry are clear: the efficacy and side effects of immunosuppressive drugs, pharmodynamic profiling, cellular functions and disease activity. However, like any other laboratory technique, also this method has its typical difficulties. A limitation is the availability of antibodies of interest. Yet it is expected that more and more phospho- and epitopic-specific antibodies will become available soon. For instance, antibodies that detect TCR-induced T-cell activation would be highly valued. These antibodies can be used to determine whether or not T cells are present in the peripheral blood of organ transplant patients that respond to donor antigen. Analysis of activated Zap-70 will provide this key information and will at the same time be the functional alternative that overcomes the difficulties and limitations of TCR tetramer flow cytometry [33]. Further, simultaneous measurements of phosphorylated proteins together with cytokines are far from optimal, and reproducibility from one experiment to the next remains a problem. In this respect, standardization of the instrument using multicolor bead samples is helpful. Data interpretation is also a question to address: should percentages or mean fluoresce intensities (MFI) be used. The relative MFI values are based on control samples like the unstained control samples, isotype control or fluorescent minus one control to set thresholds and are used to measure a shift in fluorescence intensity of the entire cell populations. Consequently, it is not clear how many cells of the entire cell population have changed expression of the molecule of interest.

As stated in the paper by Herzenberg et al., interpreting flow cytometry data is the ‘Tower of Babel’: negative, unstained populations can seem to be positive and vice versa [34]. Therefore, optimization and standardization are key for the success of (phospho-specific) flow cytometry for measuring immune responses in healthy and diseased individuals. Addressing these limitations will further improve the utility of phospho-specific flow cytometry for clinical applications.

## Conclusions

Recently, phospho-specific flow cytometry came forward as a new and powerful tool to analyze the activity of intracellular signaling pathways by rapid and sensitive detection of intracellular phosphorylated proteins at the single-cell level. It is a technique that can be used to study (pathological) conditions in immune cells, can be used as a screening tool to identify novel targets of immunosuppressive drugs and for pharmacodynamic monitoring of patients on immunosuppressive medication. In particular patients treated with drugs that target these intracellular signaling molecules: the inhibitors of calcineurin, mTOR and kinase activity should be monitored by phospho-specific flow cytometry.

Analysis of signaling pathways in large patient populations will show patient-specific differences in immune reactivity, drug susceptibility and drug-related side effects.

## Abbreviations

rATG: Rabbit anti thymocyte globulin; CNI: Calcineurin inhibitor; CTLA-4: Cytotoxic T-lymphocyte antigen-4; GFR: Glomerular filtration rate; JAK: Janus kinase; LAT: The linker for activated T cells; Lck: Lymphocyte protein-tyrosine kinase; mAb: Monoclonal antibody; MAPK: Mitogen-activated protein kinase; MFI: Mean fluoresce intensities; MMF: Mycophenolate mofetil; mTOR: Mammalian target of rapamycin; PKC: Protein kinase C; STAT: Signal transducer and activator of transcription; Treg: Regulatory T cells; TYK2: Tyrosine kinase 2.

## Competing interests

The authors declare that they have no competing interests.

## Authors’ contributions

CB designed and coordinated the study, and wrote the paper. AB participated in the research cited in this manuscript. RV participated in the research cited in this manuscript. WW participated in the writing of the manuscript, in its critical appraisal. All authors read and approved the final manuscript.
